# Intraoperative changes in electrophysiological monitoring can be used to predict clinical outcomes in patients with spinal cavernous malformation

**DOI:** 10.1515/med-2024-1008

**Published:** 2024-10-18

**Authors:** Xiaoyu Li, Hongqi Zhang, Jian Ren

**Affiliations:** Xuanwu Hospital of Capital Medical University, Beijing, China; Department of Neurosurgery, Xuanwu Hospital, Capital Medical University, China International Neuroscience Institute (China-INI), Beijing, 100053, China

**Keywords:** motor evoked potentials, electrophysiological monitoring, spinal cavernous malformations, somatosensory evoked potentials, intraoperative neuromonitoring

## Abstract

**Aim:**

The study aimed to evaluate the sensitivity and specificity of these monitoring parameters in predicting postoperative neurological dysfunction.

**Methods:**

In this study, a total of 85 patients with spinal cavernous malformations (SCMs) treated at Xuanwu Hospital, Capital Medical University, from November 2012 to August 2017 were included. During the surgical procedures, all patients underwent monitoring of motor evoked potentials (MEP) and somatosensory evoked potentials (SEP). The criteria for warning included a reduction of ≥80% in MEP amplitude and ≥50% in SEP amplitude.

**Results:**

Among 85 patients, 40 (47.1%) had SCMs located in the thoracic segment, 35 (41.2%) in the cervical segment, 6 (7.1%) in the cervical thoracic segment, and 4 (4.7%) in the lumbar segment. MEP recordings were obtained from 81 patients, and the preoperative McCormick score was 1.53 ± 0.69. The sensitivity of multimodal monitoring combined with the criteria of 80% reduction in MEP amplitude and SEP was 83.9%, with a specificity of 69%, a positive predictive value of 69%, and a negative predictive value of 90.4%.

**Conclusion:**

This study emphasizes the crucial role of electrophysiological monitoring, particularly MEP and SEP, during the surgical resection of SCMs. The findings demonstrate that this approach is effective in predicting and preventing postoperative neurological dysfunction, thereby improving patient outcomes.

## 1 Introduction

Spinal cavernous malformations (SCMs) are rare vascular lesions found within the spinal cord, primarily in the thoracic region. These SCMs represent approximately 5–12% of all spinal vascular disorders, as reported by various studies [1–3]. Due to the location of these lesions, patients may experience sensory and motor dysfunction in their lower extremities. Without timely surgical intervention, the onset of bleeding symptoms could lead to a further decline in the patient’s spinal cord function, potentially resulting in paralysis. Therefore, surgical resection is regarded as the optimal approach to prevent the incidence of bleeding. However, as the removal of intramedullary lesions may impact the sensory and motor conduction pathways, intraoperative neuromonitoring (IONM) becomes essential during the surgical procedure [4–6].

The first monitoring method used in spinal cord surgery was the somatosensory evoked potential (SEP), as reported by Engler et al. in 1978. However, SEP can only reflect the function of the spinal cord’s dorsal side, leading to potential false negatives. For instance, SEP may appear normal while patients experience motor dysfunction [7–9]. As motor function is crucial for patients’ postoperative function, the motor evoked potential (MEP) has been applied in spinal cord surgery since the 1990s [10]. In recent years, the combined application of SEP and MEP has become a routine method in spinal cord surgery. However, there is no consensus on the definition criteria for MEP, leading to reported false positives and negatives [11]. As surgical monitoring examinations deepen, studies have found that monitoring criteria may vary for different types of lesions [12].

The present study aimed to assess the results of IONM and postoperative functional changes in 85 patients with SCM. The sensitivity and specificity of different monitoring criteria were also analyzed for the combination of SEP and MEP, suggesting optimal criteria.

## 2 Methods

### 2.1 Patients’ data

This study included 85 patients with SCM who were admitted to our hospital from November 2012 to August 2017, including 43 men and 42 women, with an average age of 38.8 ± 13.2 (range, 13–73) years old. The lesion segmentation was determined according to the imaging results. All patients underwent neurological assessment before surgery, 3 h after surgery, at discharge, and 3–6 months after discharge. All surgeries were performed by an experienced surgeon.


**Ethical approval:** The study was approved by the Ethics Committee of the Xuanwu Hospital (approval no. [2019] 044) and was conducted in accordance with the 1964 Helsinki Declaration and its later amendments or comparable ethical standards. Informed consent was waived by our Institutional Review Board because of the retrospective nature of our study.

### 2.2 IONM

The IONM was performed using the Nicolet System (Natus Neuro, Middleton, WI, USA). Neuromonitoring was conducted by the same surgical team throughout the procedure. Technicians were responsible for electrode placement, while a neurologist with over 10 years of monitoring experience confirmed baseline readings and interpreted intraoperative changes. The IONM data were then reviewed by two independent observers after surgery.

### 2.3 MEP

According to the lesion segmentation, the corresponding muscle was selected to record MEP. The abductor pollicis brevis was selected for the upper limb, and the abductor brevis, anterior tibialis, or quadriceps femoris were selected for the lower limb. A pair of needle electrodes were placed to record MEP. The stimulation point was located according to the 10–20 electroencephalogram (EEG) system, which is based on the relationship between the location of an electrode and the underlying area of the brain, specifically the cerebral cortex. C1 and C2 were selected to, respectively, place spiral electrodes to stimulate each other. Five trains were used for each stimulation, and the wave width was 300 μs. The intensity of stimulation was 150–400 V. Each stimulation was conducted after notifying the surgeon. Gauze or dental pads were placed between the teeth and the tongue in the patient’s mouth to prevent tongue biting injury as a result of masseter contraction during stimulation.

### 2.4 SEP

Two needle electrodes were placed in the median nerve of the upper limb for stimulation, and the stimulation parameter consisted of a wave width of 300 μs. Continuous stimulation was used, with stimulation frequency of 4.7 Hz and stimulation intensity of 10–20 mA. Two needle electrodes were placed in the posterior tibial nerve of the lower limbs for stimulation. The recording position still follows the 10–20 EEG system. The recording points of the lower limbs were 1–2 cm after the Cz and Fz for reference, and the recording points of the upper limbs were 1–2 cm after the C3 and C4, and Fz for reference.

### 2.5 Electromyography (EMG) recording

Free-running EMG was recorded using the same channels used for MEP for segmental and root recordings.

### 2.6 Anesthesia

Anesthesia induction was achieved by administering intravenous midazolam (1–2 mg), etomidate (0.15 mg/kg), sufentanil (0.3 μg/kg), rocuronium (0.6 mg/kg), or atracurium (0.15 mg/kg). After induction, muscle relaxants were no longer used. Remifentanil (0.2–0.4 μg/kg/min) and propofol (4-6 mg/kg/h) were injected for anesthesia maintenance.

The bispectral index was intraoperatively used to continuously monitor the depth of anesthesia, and the depth of anesthesia was kept stable during the removal of lesions.

### 2.7 Relationship between IONM and postoperative neurological function

True-positive: The IONM assessment was in line with the newly observed postoperative neurological deficit.

True-negative: No warning was found by the IONM, and there was no loss of neurological function after the surgery.

False-negative: No warning was found by the IONM; however, there was a new postoperative neurological deficit.

False-positive: Warning was detected by the IONM, while there was no new postoperative neurological deficit.

In the present study, the reduction of McCormick grade by 1 or more was indicative of a new neurological deficit.

### 2.8 Statistical analysis

Patients’ data were analyzed to determine the relationship between the monitoring capability of SEP and MEP and postoperative results, in which age, gender, and spinal cord injury level were considered. The Chi-square test was used to evaluate the null hypothesis, and there was no correlation between categorical variables and monitoring results. If the sample size was less than or equal to five, the Fisher’s exact test was utilized. The sensitivity, specificity, positive predictive value (PPV), and negative predictive value (NPV) of different monitoring standards for prediction of postoperative neurological function were calculated. The statistical analysis was performed using SPSS 16.0 software (IBM, Armonk, NY, USA). A two-sided *P* < 0.05 was considered statistically significant.

## 3 Results

### 3.1 Location of SCM and the monitorability of MEP

Table 1 shows patients’ baseline demographic characteristics. Among 85 patients, there were 40 (47.1%), 35 (41.2%), 6 (7.1%), and 4 (4.7%) patients with SCM located in the thoracic segment, cervical segment, cervical thoracic segment, and lumbar segment, respectively. In 81 patients, MEP could be recorded, and the preoperative McCormick score was 1.53 ± 0.69. In four patients whose MEP could not be recorded, preoperative McCormick score was 4 in one patient, and it was 5 in the other three patients. The mean preoperative McCormick scores were 1.49 ± 0.64 and 2.30 ± 1.49 for SEP-measurable and non-measurable patients, respectively.

**Table 1:** Patients’ baseline characteristics

Age (years), average	38.8 ± 13.2
**Gender,** ***n*** **(%)**	
Male	43 (50.6%)
Female	42 (49.4%)
Total	85
**Lesions site,** ***n*** **(%)**	
Cervical	35 (41.2%)
Cervicothoracic	6 (7.1%)
Thoracic	40 (47.1%)
Lumbar	4 (4.7%)
Total	
**Modified McCormick grade at admission,** ***n*** **(%)**	
I	46 (54.1%)
II	28 (32.9%)
III	6 (7.1%)
IV	2 (2.4%)
V	3 (3.5%)
Total	85

### 3.2 Relationship between IONM findings and postoperative neurological function

#### 3.2.1 MEP

Table 2 presents patients’ postoperative neurological function for occurrence of all single evoked potential warning changes. The two criteria of 80% amplitude reduction and amplitude disappearance were analyzed. Temporary changes and irreversible changes were analyzed, respectively. When 80% amplitude reduction was used as the MEP criterion, 27 (33.3%) patients exhibited MEP changes intraoperatively, of whom 7 (8.2%) and 20 (23.5%) patients were found with temporary changes and irreversible changes, respectively. These seven patients with temporary changes did not have postoperative dysfunction, while among 20 patients with irreversible MEP changes, 13 patients had postoperative dysfunction, of whom 4 patients had dysfunction immediately after surgery, 7 had dysfunction at discharge, and 2 had dysfunction at follow-up.

**Table 2:** Changes in IONM and postoperative outcomes

Clinical status	Monitoring status											
	No change			Temporary changes			Permanent changes			Total		
	SEP	tcMEP (disappeared)	tcMEP (>80% reduction)	SEP	tcMEP (disappeared)	tcMEP (>80% reduction)	SEP	tcMEP (disappeared)	tcMEP (>80% reduction)	SEP	tcMEP	Any
	52	*n* = 69	*n* = 54	*n* = 2	4	7	11	8	20	65	81	85
Stable	44	50	42	2	4	7	2	2	7	48	56	60
Deficit immediately after surgery	8	19	12	0	0	0	9	6	13	17	25	25
Deficit at hospital discharge	3	11	7	0	0	0	7	3	7	8	14	14
Deficit at long-term follow-up	0	6	4	0	0	0	6	0	2	4	6	6

When using the amplitude disappearance as the warning criterion of MEP, a total of 12 (14.8%) patients had intraoperative changes, of whom four patients had temporary changes and eight patients had irreversible changes. These four patients with temporary changes had no postoperative dysfunction, whereas six of eight patients with irreversible changes had postoperative dysfunction, of whom three patients had dysfunction at discharge. At follow-up, the dysfunction of these patients was recovered.

#### 3.2.2 SEP

The warning criterion of SEP is when the amplitude is reduced by 50% or when the latency is prolonged by 10%. The results of intraoperative SEP changes and postoperative clinical function are summarized in Table 2. No patient with intraoperative SEP latency changes was found in this study; thus, patients with SEP changes were those with the reduced amplitude. Among 65 patients whose SEP could be recorded, there were two patients with temporary changes and 11 patients with irreversible changes. Of 11 patients, 6 patients had postoperative functional changes, 2 had dysfunction at discharge, and 3 had dysfunction at follow-up.

#### 3.2.3 Multimodality IONM correlation analysis

The relationship between any changes in MEP and clinical outcomes is presented in Table 3 (>50% reduction of MEP) and Table 4 (disappearance of MEP).

**Table 3:** Relationship between any changes in evoked potential and clinical outcomes (>50% reduction in SEP or >80% reduction in tcMEP)

Clinical status	Monitoring status (>80% reduction tcMEP)			
	No change	Temporary changes	Permanent changes	Total
	43	9	29	81
Stable	38	9	9	56
Deficit immediately after surgery	5	0	20	25
Deficit at hospital discharge	2	0	12	14
Deficit at long-term follow-up	0	0	6	6

**Table 4:** Relationship between any changes in evoked potential and clinical outcomes (>50% reduction in SEP or disappearance of tcMEP)

Clinical status	Monitoring status (disappearance of MEP)			
	No change	Temporary changes	Permanent changes	Total
	57	5	19	81
Stable	47	5	4	56
Deficit immediately after surgery	10	0	15	25
Deficit at hospital discharge	3	0	10	13
Deficit at long-term follow-up	0	0	6	6

Table 3 shows the relationship between 80% amplitude reduction as the warning criterion of MEP and postoperative function, combined with multimodal SEP monitoring. Besides, six patients had temporary changes intraoperatively, and no patient had postoperative dysfunction. Moreover, 29 patients had irreversible changes of MEP, of whom 20 patients had postoperative dysfunction. Among 20 patients with postoperative dysfunction, 12 had dysfunction at discharge and 6 had dysfunction at follow-up.

Table 4 shows the relationship between the disappearance of wave amplitude as the warning criterion of MEP combined with multimodal SEP monitoring and postoperative function. It was revealed that five patients had temporary changes in intraoperative MEP, and no patient had postoperative dysfunction. Furthermore, 19 patients had irreversible changes in MEP, of whom 15 had postoperative dysfunction. Among 15 patients, 10 had dysfunction at discharge and 6 had functional impairment after discharge.

#### 3.2.4 Sensitivity and specificity analyses

The results of sensitivity and specificity analyses are presented in Table 5. According to the proposed criteria, patients’ prognosis was determined as true-positive, true-negative, false-positive, or false-negative.

**Table 5:** Sensitivity and specificity of both permanent IONM changes

	>80% reduction in tcMEP	Disappearance of tcMEP
True negative (*n*)	47	52
True positive (*n*)	20	15
False negative (*n*)	5	10
False positive (*n*)	9	4
Sensitivity (%)	80	60.0
Specificity (%)	83.9	92.9
Positive predicting result (%)	69.0	78.9
Negative predicting result (%)	90.4	83.9

According to the results summarized in Tables 2–4, patients with temporary changes in SEP or MEP did not have postoperative dysfunction. Therefore, temporary changes were not involved in the sensitivity and specificity analyses. Regarding the sensitivity of multimodal monitoring combined with 80% MEP amplitude reduction and SEP, the specificity was 83.9%, the PPV was 69%, and the NPV was 90.4%. Taking the disappearance of MEP amplitude as the warning criterion combined with the results of the SEP, the sensitivity, specificity, PPV, and NPV were 60.0, 92.9, 78.9, and 83.9%, respectively.

#### 3.2.5 Case presentation

##### 3.2.5.1 Case 1

**Figure 1:**
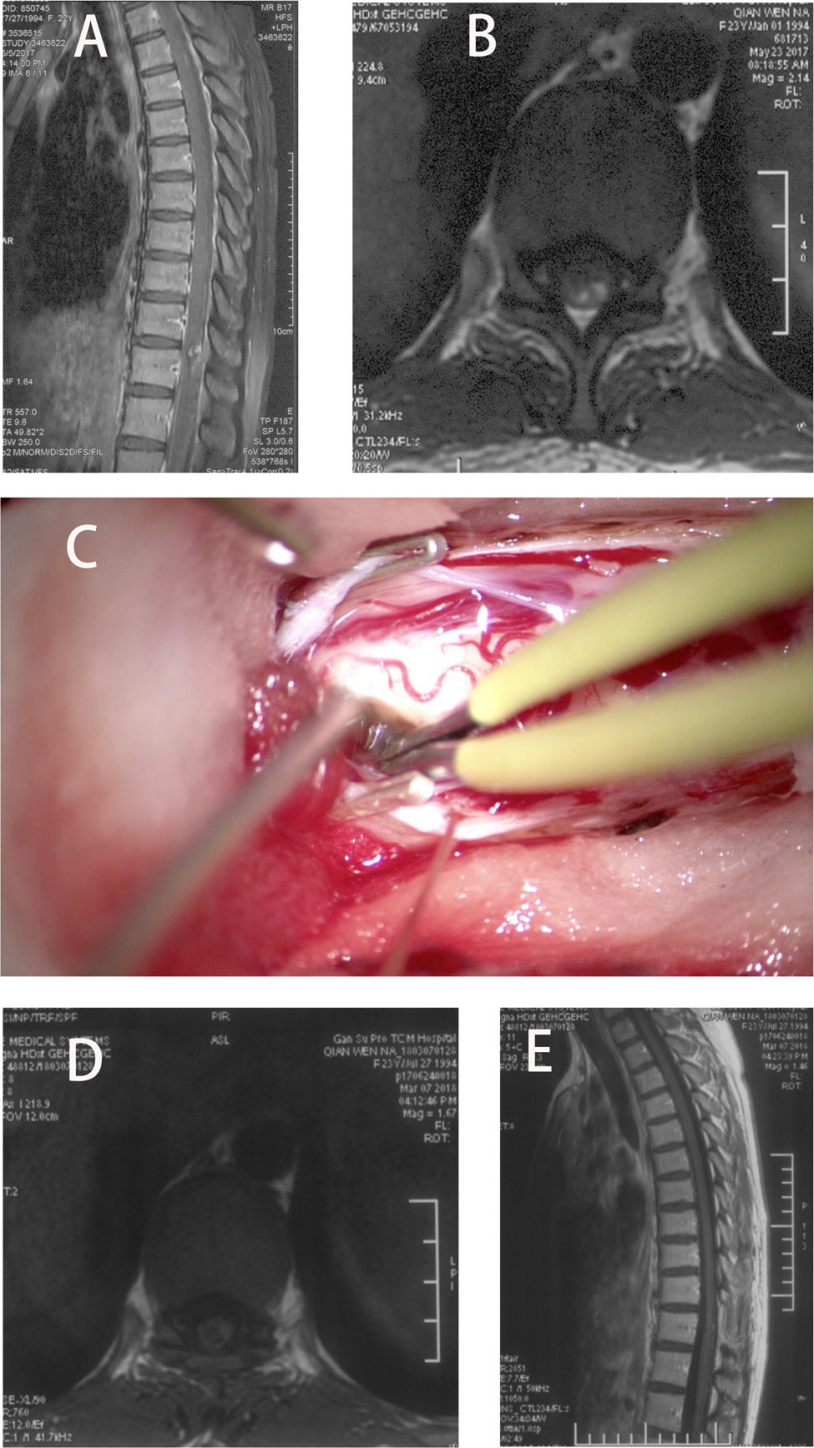
Preoperative MRI (a) and (b), and intraoperative findings (c) showed an SCM at T10–T12 level. Postoperative MRI (d) and (e).

**Figure 2:**
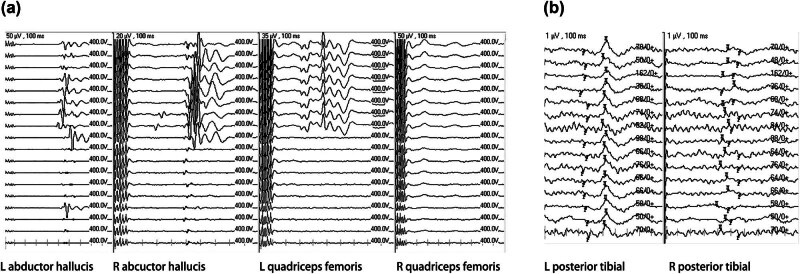
A 22-year-old woman with a history of pain for 52 days, accompanied by dysuria and decreased lower limb muscle strength (3/5 of the left lower limb and 4/5 of the right lower limb). The patient’s McCormick score was grade 3. SEP (b) and MEP (a) of both lower limbs were monitored intraoperatively. Additional surgical findings and conditions are presented in Section 3.2.5.

A 22-year-old woman was admitted to our hospital with history of pain for 52 days, accompanied by dysuria and decreased lower limb muscle strength (3/5 of the left lower limb and 4/5 of the right lower limb). Magnetic resonance imaging (MRI) revealed that lesions extended from T10 to T11 vertebral bodies, and she was diagnosed with SCM (Figure 1). The patient’s McCormick score was 3. In addition, SEP and MEP of both lower limbs were monitored intraoperatively (Figure 2). During the surgery, MEP of abductor hallucis and quadriceps femoris decreased by more than 80%, and MEP of abductor pollicis brevis muscle disappeared (the baseline MEP of the right quadriceps femoris was not involved). After elimination of the technical failure, the surgery was suspended. After 30 min, the left MEP returned to more than 50% of the baseline, and the surgeon decided to continue the surgery. The surgeon attempted to minimize interference with the spinal cord as much as possible during the ongoing resection. However, at the end of the lesion resection, the MEP of the left abductor pollicis brevis and quadriceps femoris was reduced by more than 80% again. The patient’s SEP was stable throughout the surgery. We locally applied papaverine and increased the mean arterial pressure to over 90 mmHg to resist local ischemia. After the surgery, the right leg muscle strength was grade 2, the left leg muscle strength was grade 3, and McCormick score was 2. She was recovered to the preoperative level at 3 months after the surgery. The patient was defined as true-positive for the monitoring results.

##### 3.2.5.2 Case 2

**Figure 3:**
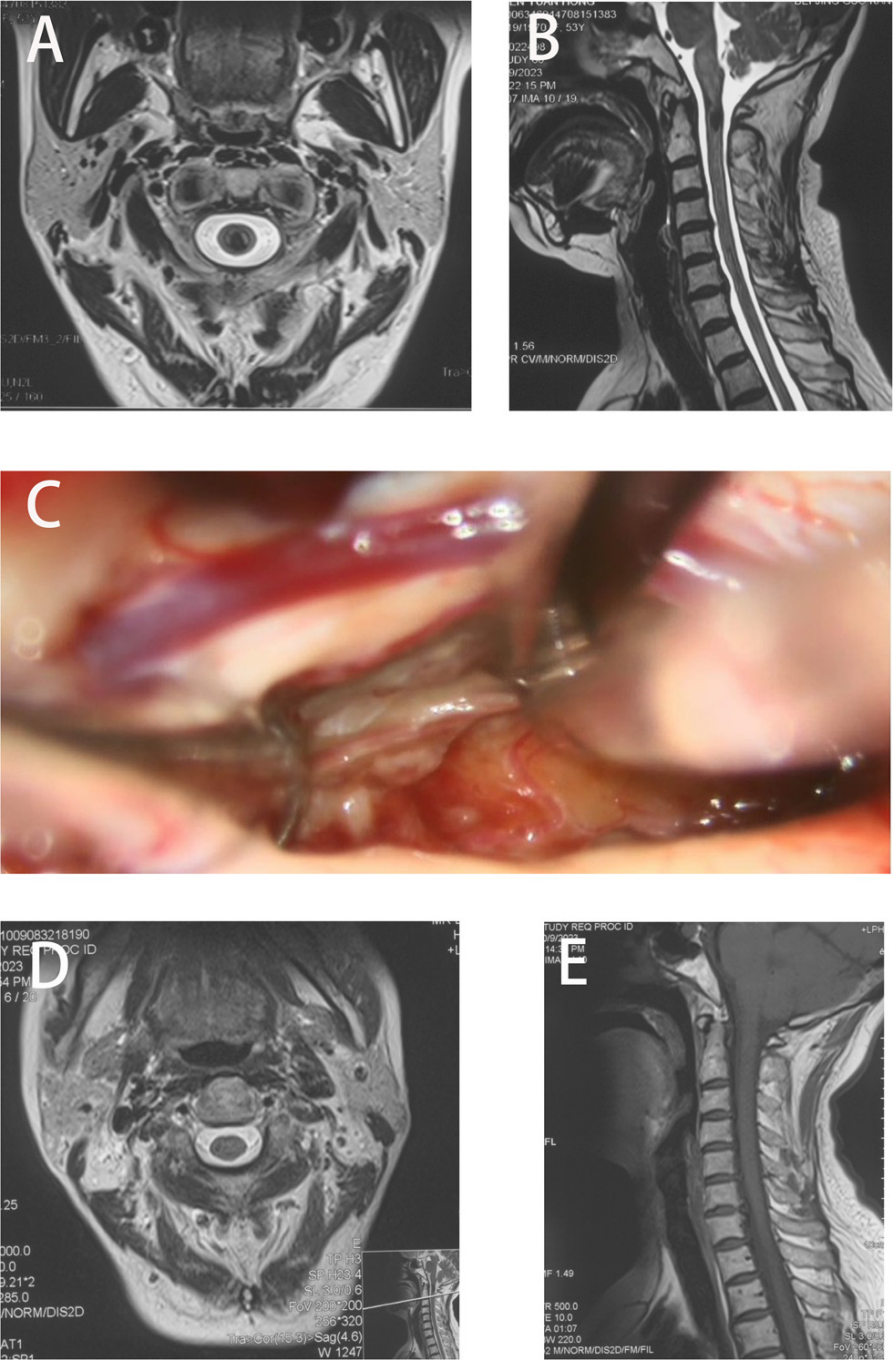
Preoperative MRI (a) and (b), and intraoperative findings (c) showed an SCM at C1–C2 level. Postoperative MRI (d) and (e).

**Figure 4:**
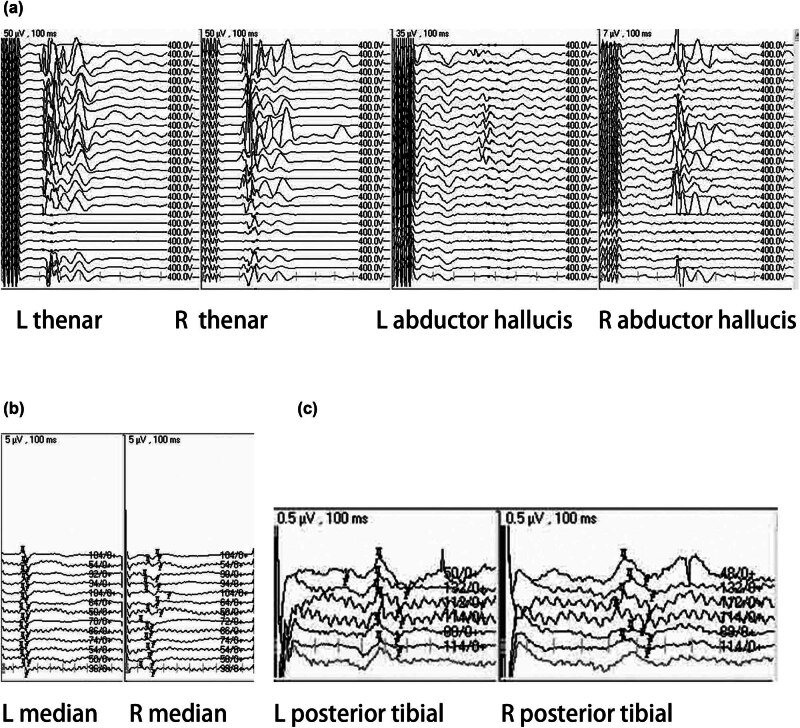
A 54-year-old woman was admitted for surgical treatment due to a cervical lesion discovered during a physical examination. Prior to surgery, the patient had numbness in both upper limbs, normal sensation in both lower limbs, and normal muscle strength in all limbs. During the surgery, the SEP (b) and (c) and MEP (a) in both upper and lower limbs were monitored. Additional surgical findings and conditions are presented in Section 3.2.5.

A 54-year-old woman was admitted for surgical treatment due to a cervical lesion discovered during a physical examination (Figure 3). Prior to surgery, the patient had numbness in both upper limbs, normal sensation in both lower limbs, and normal muscle strength in all limbs. During the surgery, the SEP and MEP in both upper and lower limbs were monitored. When the lesion was removed during surgery, the patient’s MEP in both upper and lower limbs decreased by more than 80%. The surgeon was reminded to pause the surgery and administer glucocorticoid and warm saline to rinse the surgical area. After the evoked potentials recovered by more than 50%, surgery continued, and the patient had no new postoperative functional impairment (Figure 4).

##### 3.2.5.3 Case 3

**Figure 5:**
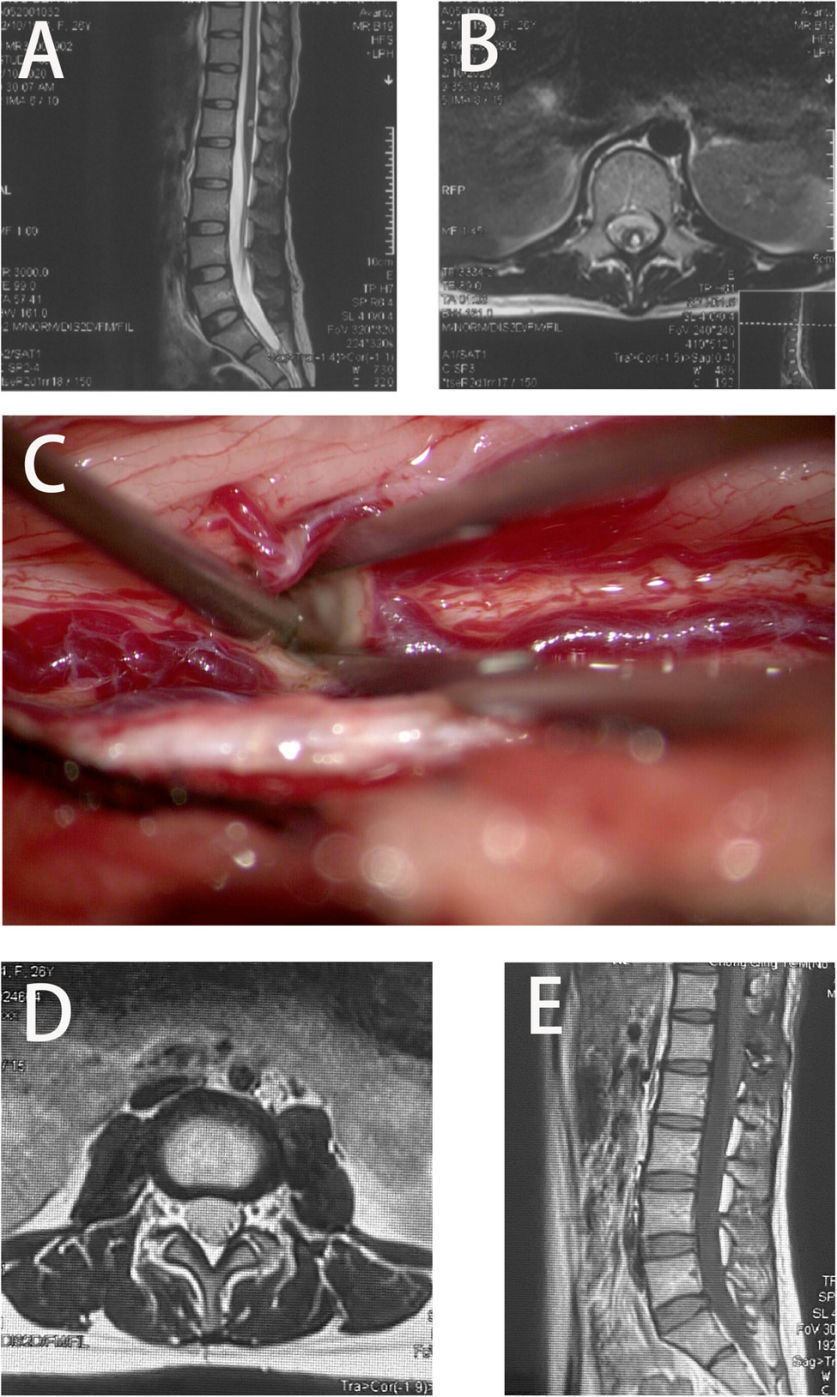
Preoperative MRI (a) and (b), and intraoperative findings (c) showed an SCM at L1 level. Postoperative MRI (d) and (e).

**Figure 6:**
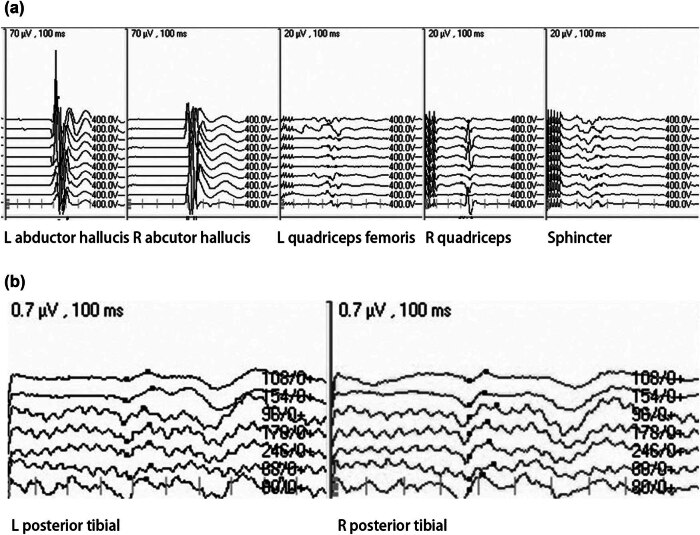
A 26-year-old female patient presented with bilateral lower limb numbness and lower back pain for over 3 months. An MRI revealed a spinal cord cavernous malformation at the L1 level. Intraoperatively, SEP (b) of the tibial nerve in both lower limbs and MEP (a) of the proximal and distal muscles of both lower limbs, as well as the anal sphincter, were monitored. No significant decrease in sensory evoked potential or MEP was observed during tumor resection. Following the surgery, the patient did not experience worsening sensory deficits or a decline in muscle strength.


A 26-year-old female patient presented with bilateral lower limb numbness and lower back pain for over 3 months. MRI revealed a spinal cord cavernous malformation at the L1 level (Figure 5). The patient presented with numbness in both lower limbs and a muscle strength grade of 5- in both lower limbs on preoperative examination. Intraoperatively, SEP of the tibial nerve in both lower limbs and MEP of the proximal and distal muscles of both lower limbs, as well as the anal sphincter, were monitored. During tumor resection, there was no significant decrease in sensory or MEPs (Figure 6). Postoperatively, the patient did not experience worsening sensory deficits or a decline in muscle strength.

**Informed consent:** Informed consent was obtained from the patients.

## 4 Discussion

SCM is a relatively rare disease. Because of its special location and close relationship with the blood supply of the spinal cord, it is easy to cause damage to the spinal cord during resection, resulting in postoperative dysfunction. Therefore, IONM of MEP is essential [13]. To our knowledge, this is the largest study that performed electrophysiological monitoring on patients with SCM. The present study analyzed the results of IONM and patients’ neurological function after surgery of SCM, and it compared the changes of postoperative function with intraoperative 80% amplitude reduction and disappearance of MEP, which were utilized as the warning criteria. When 80% amplitude reduction was used as the warning criterion, combined with SEP, the sensitivity was 80%, the specificity was 83.9%, the PPV was 69%, and the NPV was 90.4%. Taking the disappearance of MEP amplitude as the warning criterion, combined with the multimodal monitoring results of SEP, the sensitivity was 60.0%, the specificity was 92.9%, the PPV was 78.9%, and the NPV was 83.9%.

The routine use of both MEP and SEP in multimodal monitoring during spinal cord surgery has been promoted. However, the warning criterion of MEP has been frequently applied in various studies [14,15]. The most conventional monitoring criteria include 50% amplitude reduction, 80% amplitude reduction, amplitude disappearance, and reversible and irreversible changes. With the deepening of monitoring experience, the monitoring criteria need to be extensively discussed. Monitoring results may vary for lesions located in different segments, or even for lesions with distinct properties within the same segment. Consequently, the criteria for monitoring alerts may also differ [16]. Our research group has previously compared the monitoring results of spinal arteriovenous malformation and SCM [17], and found that there were significant differences between them. Therefore, it is essential to summarize the monitoring results of a specific surgery. In the present study, for surgery of SCM, it was found from the monitoring results of the MEP that temporary changes in MEP could not cause postoperative dysfunction. However, a 50% amplitude reduction was used as the criterion, which exhibited no difference from the 80% amplitude reduction. The disappearance of this warning criterion was associated with high specificity, while it produced more false negative results, and false negative results are the most important problem in monitoring. Therefore, in clinical practice, the disappearance of MEP is a more suitable standard for suspending surgery, whereas reductions of 50 and 80%, and SEP reductions over 50%, are more appropriate as standards for alerting the surgeons. Following these standards, it is advisable to proceed with surgery as gently as possible and provide warm saline and steroids in the surgical area. In the case of surgery for spinal cord cavernous vascular malformation, different degrees of IONM warnings have significance for the surgeon, but the treatments should correspond accordingly.

In the present study, all patients with temporary changes did not have postoperative disorders, including two patients with SEP amplitude reduction and seven patients with MEP amplitude reduction of more than 80% (the amplitude disappeared in four patients). This indicates that if warning criteria appear intraoperatively, and corresponding treatment measures are taken such that the induced potential amplitude can be restored, patients may generally not experience postoperative dysfunction. Therefore, these results also underscore the importance of timely intervention when warning signs in MEP are observed.

The MEP may serve as a nonlinear and unstable indicator. After reaching the appropriate threshold of stimulation, the amplitude of the MEP does not increase due to the enhancement of stimulation, and even if the recorded muscle strength is normal, the amplitude of the MEP may not be completely consistent. Therefore, during intraoperative monitoring, it is crucial to not only use staged warning criteria, but also to understand that the trend of variations is more significant than a single variation. This is because frequent warnings due to individual variations of MEP may impact the normal course of the surgery. In addition to investigating the correlation between MEP warning criteria and postoperative neurological function, it is also necessary to identify which types of surgeries are more likely to result in changes to MEP [18].

Few surgeons may consider an individual reduction of SEP as a criterion to halt surgery. Therefore, in the current spinal cord surgeries, SEP can be primarily used as a reference in conjunction with MEP. Moreover, a remarkable number of patients with spinal cord lesions have preoperative sensory impairment, resulting in an unsatisfactory preoperative rate of SEP. In this study, SEP was feasibly recorded preoperatively in 65 patients, with an elicitation rate of 76.5%, while the elicitation rate of MEP was 95.3%. Among 11 patients with irreversible SEP changes, 9 patients experienced postoperative dysfunction, including 7 patients with only SEP changes who had postoperative sensory dysfunction. However, the long-term recovery effect on patients with sensory impairment was suboptimal, with the majority of patients with long-term dysfunction primarily experienced numbness. Therefore, changes in SEP are of significant importance.

The false negative rate in this study was higher than previously reported rate [19,20], which could be related to strict definition of neurological dysfunction in the present study. Patients whose McCormick score was reduced by 1 were included in the dysfunction category. False negative reports of SEP combined with MEP were very limited and controversial in the recent decade. According to the 80% MEP reduction criterion, combined with the multimodal monitoring of SEP, five patients with false negative results were found. Among five patients, the McCormick scores of four patients were only reduced by 1 point. Postoperative dysfunction was mainly the reduction of muscle strength by 1 point or the emergence of new postoperative sensory dysfunction. Only one patient whose McCormick score was reduced by 2 points had a 50% amplitude reduction of MEP; however, this did not meet the warning criteria used in this study. Among five patients, one lesion was located in the cervical segment, and the other four lesions were located in T10–T12 (close to the conus medullaris), indicating that there may be a blinded spot of neuromonitoring during the lesion resection. Among nine patients with false positive results, lesions in six patients were located in the cervical segment or cervical thoracic segment, demonstrating that the cervical spinal cord was swiftly recovered.

There were some limitations in this study. First, because the incidence rate of SCM was low, the sample size was not large enough. Second, as the electrodes placed in the spinal cord were not licensed in China, no intraoperative D wave was applied in the monitorability and motor outcome in spinal surgery. Finally, no control group was established.

## 5 Conclusions

In conclusion, the combination of SEP and MEP for intraoperative monitoring during surgical removal of the SCM can effectively prevent postoperative neurological dysfunction. Warning criteria for MEP can be based on staged amplitude reductions, with a threshold typically set at an 80% reduction. When removing intraspinal space-occupying lesions, various monitoring systems and early warning criteria may have distinct characteristics based on the location and type of lesion.
